# Early Changes in Alpha Band Power and DMN BOLD Activity in Alzheimer’s Disease: A Simultaneous Resting State EEG-fMRI Study

**DOI:** 10.3389/fnagi.2017.00319

**Published:** 2017-10-06

**Authors:** Katharina Brueggen, Carmen Fiala, Christoph Berger, Sina Ochmann, Claudio Babiloni, Stefan J. Teipel

**Affiliations:** ^1^German Center for Neurodegenerative Diseases, Rostock, Germany; ^2^Department of Psychosomatic Medicine, University Medicine Rostock, Rostock, Germany; ^3^Department of Psychiatry, Neurology, Psychosomatics, and Psychotherapy in Childhood and Adolescence, University of Rostock, Rostock, Germany; ^4^Department of Physiology and Pharmacology “Vittorio Erspamer”, University of Rome “La Sapienza”, Rome, Italy; ^5^Department of Neuroscience, IRCCS San Raffaele Pisana, Rome, Italy

**Keywords:** Alzheimer’s disease, alpha rhythm, electroencephalography, functional magnetic resonance imaging, default mode network

## Abstract

Simultaneous resting state functional magnetic resonance imaging (rsfMRI)–resting state electroencephalography (rsEEG) studies in healthy adults showed robust positive associations of signal power in the alpha band with BOLD signal in the thalamus, and more heterogeneous associations in cortical default mode network (DMN) regions. Negative associations were found in occipital regions. In Alzheimer’s disease (AD), rsfMRI studies revealed a disruption of the DMN, while rsEEG studies consistently reported a reduced power within the alpha band. The present study is the first to employ simultaneous rsfMRI-rsEEG in an AD sample, investigating the association of alpha band power and BOLD signal, compared to healthy controls (HC). We hypothesized to find reduced positive associations in DMN regions and reduced negative associations in occipital regions in the AD group. Simultaneous resting state fMRI–EEG was recorded in 14 patients with mild AD and 14 HC, matched for age and gender. Power within the EEG alpha band (8–12 Hz, 8–10 Hz, and 10–12 Hz) was computed from occipital electrodes and served as regressor in voxel-wise linear regression analyses, to assess the association with the BOLD signal. Compared to HC, the AD group showed significantly decreased positive associations between BOLD signal and occipital alpha band power in clusters in the superior, middle and inferior frontal cortex, inferior temporal lobe and thalamus (*p* < 0.01, uncorr., cluster size ≥ 50 voxels). This group effect was more pronounced in the upper alpha sub-band, compared to the lower alpha sub-band. Notably, we observed a high inter-individual heterogeneity. Negative associations were only reduced in the lower alpha range in the hippocampus, putamen and cerebellum. The present study gives first insights into the relationship of resting-state EEG and fMRI characteristics in an AD sample. The results suggest that positive associations between alpha band power and BOLD signal in numerous regions, including DMN regions, are diminished in AD.

## Introduction

In Alzheimer’s disease (AD), resting state functional magnetic resonance imaging (rsfMRI), and resting state electroencephalography (rsEEG) have only been used separately to measure pathological changes. RsfMRI studies showed decreased activity (Greicius et al., 2004; Zhu et al., 2013; Li et al., 2015) and disrupted functional connectivity (Greicius et al., 2004; Zhang et al., 2009, 2010; Agosta et al., 2012; Koch et al., 2012; Weiler et al., 2014; Xia et al., 2014) in the default mode network (DMN) in AD. The DMN includes the anterior and posterior cingulate cortex, precuneus, medial prefrontal cortex, inferior parietal cortex, and hippocampal formation (Shulman et al., 1997; Raichle et al., 2001; Greicius et al., 2003; Buckner et al., 2008). Functionally, it has been associated with episodic memory (Mazoyer et al., 2001; Buckner et al., 2008; Weiler et al., 2014) and self-referential thinking (Raichle et al., 2001; Greicius et al., 2004; Buckner et al., 2008; Knyazev, 2013). Furthermore, rsEEG analyses showed reduced power within the alpha band (8–12 Hz) at early AD stages, as well as a slowing of the alpha rhythm and increased presence of lower frequency bands (Brenner et al., 1986; Dierks et al., 1993; Huang et al., 2000; Jeong, 2004). The alpha band is the dominant rhythm in healthy adults during a state of relaxed wakefulness, keeping the eyes closed (Berger, 1929; Zschocke and Hansen, 2012; Hinrichs, 2015). It originates from thalamo-cortical neurons projecting to the occipital cortex (Lorincz et al., 2009; Hughes et al., 2011; Zschocke and Hansen, 2012; Babiloni et al., 2015) – a projection pathway that may be disrupted in AD, as shown previously in studies using a computational model (Bhattacharya et al., 2011) and fMRI functional connectivity (Zhou et al., 2013). Functionally, alpha band power was shown to correlate positively with internal mental processes (Knyazev et al., 2011). Moreover, subdivisions of the alpha band may be related to different cognitive functions: the lower alpha band (8–10 Hz) may be associated with attention, while the upper alpha band (10–12 Hz) may be associated with memory processes (Klimesch, 1999). In addition, alpha band power has been suggested to play a role in an inhibitory gating mechanism of the visual system, suppressing unattended visual information (Berger, 1929; Palva and Palva, 2007; Tuladhar et al., 2007; Zumer et al., 2014). Power within the alpha band has been shown to correlate negatively with hemodynamic activity in the occipital cortex (Goldman et al., 2002; Moosmann et al., 2003; Gonçalves et al., 2006; Mantini et al., 2007; Scheeringa et al., 2012).

In order to assess the temporal association within subjects, the two modalities need to be measured simultaneously. The simultaneous rsfMRI-rsEEG measurement allows investigating the correlation of the BOLD signal fluctuation (as measured with rsfMRI) with the power fluctuation in specific frequency bands (as measured with rsEEG) over time. This method has previously been applied in young healthy subjects, correlating power fluctuations within the alpha band with BOLD signal fluctuations within each voxel. Most of these studies found that alpha band power fluctuation correlated positively with BOLD signal fluctuations in the thalamus (Goldman et al., 2002; Moosmann et al., 2003; Gonçalves et al., 2006) and in cortical DMN regions (Mantini et al., 2007; Jann et al., 2009, 2010; Scheeringa et al., 2012). On the other hand, some studies reported only weak or no positive associations (Laufs et al., 2003a; Gonçalves et al., 2006; Mo et al., 2013). Negative associations were found between alpha band power fluctuation and BOLD signal fluctuation in occipital, parietal, and frontal cortical regions in young HC subjects (Goldman et al., 2002; Laufs et al., 2003a, 2006; Moosmann et al., 2003; Gonçalves et al., 2006; Mantini et al., 2007; Scheeringa et al., 2012).

The present study is the first to employ simultaneous fMRI-EEG measurement in AD patients. Its aim was to explore its feasibility and to investigate the relationship of alpha band power fluctuation and BOLD signal fluctuation in AD patients compared to HC subjects. As previous research showed alpha band power to correlate significantly with gray matter volume in AD (Babiloni et al., 2009, 2013, 2015), we additionally controlled for volume of the hippocampus, which is affected early in the disease (Devanand et al., 2007; den Heijer et al., 2010; Frisoni et al., 2010; Jack et al., 2011). We hypothesized to find positive associations between occipital alpha band power fluctuation and BOLD signal fluctuation in regions of the DMN in both groups (AD and HC), with a reduced association in the AD group. Secondly, we hypothesized to find positive associations of alpha band power fluctuation and BOLD signal fluctuation in the thalamus in both groups, but a weaker association in AD. Finally, we expected to find negative associations with BOLD signal fluctuation in the occipital cortex, with reduced associations in the AD group (Moretti, 2004).

## Materials and Methods

### Participants

The groups consisted of *n* = 14 individuals each, matched for age and gender (see **Table 1** for demographic and clinical characteristics). Initially, *n* = 18 patients with mild AD and *n* = 17 elderly healthy control (HC) subjects participated in the study, of which one patient aborted the scan session, and three patients were excluded due to radiological abnormalities. Three female participants in the HC group were randomized out, in order to match the groups for gender. Patients were recruited via the memory clinic at the University Medicine Rostock (UMR); HC subjects were recruited via the database of the UMR, containing healthy subjects who were originally recruited via advertisement. HC were required to score within one standard deviation on all subscales of the Consortium to Establish a Registry for Alzheimer’s Disease (CERAD) battery (Morris et al., 1989). Patients were clinically diagnosed with probable AD dementia according to the NINCDS-ADRDA and NIA-AA criteria (McKhann, 1984; McKhann et al., 2011). All subjects underwent general medical, neurological and psychiatric assessment. Neuropsychological assessment was conducted using the CERAD battery. Laboratory analyses and APOE genotype sequencing were carried out. Subjects exhibited no neurological or radiological abnormalities (e.g., normal pressure hydrocephalus or extensive microinfarcts), and no psychiatric diseases. AD patients showed no signs of dementia not due to AD (e.g., vascular dementia). The study was approved by the local ethics committee of the University Rostock. All participants gave written informed consent, and all procedures were carried out in accordance with the Helsinki declaration.

**Table 1:** Demographic and clinical characteristics of the study subjects; mean ± SD (range).

	AD (*n* = 14)	HC (*n* = 14)	*p*^∗^
Age	75.3 ± 5.7 (64–82)	73.4 ± 3.1 (68–79)	0.276
Gender (male/female)	10/4	10/4	n. a.
Education (years)	14.4 ± 2.7 (8–17)	13.6 ± 2.8 (11–20)	0.417
MMSE score	24.6 ± 3.1 (17–28)	28.7 ± 0.8 (27–30)	<0.001
APOE status (E2/E3; E2/E4; E3/E3; E3/E4; E4/E4)	0; 2; 4; 6; 1	2; 1; 7; 2; 1	n. a.


*^∗^Independent samples *t*-test, 2-sided.*

### Data Acquisition

Electroencephalography and fMRI data were recorded simultaneously during 7.5 min of resting state (eyes-closed). For the EEG recording, MRI-compatible measurement devices (Brain Products, Gilching, Germany) and the software Brain Vision Recorder^1^ were used. EEG was recorded at 32 electrodes that were positioned according to the international 10-20-system (Jasper, 1958). The reference electrode was located between Fz and Cz, the ground electrode at AFz. Impedances of the electrodes of interest (O1, O2, and Oz) were kept below 8 kΩ, except for one AD patient (18 kΩ). An additional ECG channel was attached to detect cardio-ballistic artifacts. EEG data were sampled at 5 kHz. The EEG amplifier sampling interval was phase-synchronized to the fMRI main frequency via the Syncbox (Brain Products, Gilching, Germany) in order to preclude EEG-fMRI-sampling-jitter artifacts. The EEG hardware (i.e., amplifier and powerpack) was placed at the head end of the scanner tube and weighted with sand bags to prevent hardware motion.

Functional magnetic resonance imaging images were acquired using a 3-Tesla Siemens Magnetom scanner with a T2-weighted echo-planar imaging sequence (TR: 2.6 s, TE: 30 ms, FOV: 224 mm, thickness: 3.5 mm, number of slices: 180). The anatomical images were recorded using a T1-weighted MPRAGE sequence (TR: 2.5 s, TE: 4.37 ms, FOV 256 mm, thickness: 1 mm, number of slices: 192). Foam wedges were used to stabilize the head. Subjects were instructed to stay awake, keeping their eyes closed. The EEG signal was visually controlled for signs of sleep (offline).

### Data Preprocessing

#### EEG Data

Data were preprocessed using Brain Vision Analyzer software (Version 2.0, Brain Products, Gilching, Germany). First, data were downsampled to 250 Hz. Imaging and ECG pulse artifacts were eliminated using the average artifact subtraction method described by Allen et al. (1998, 2000), which is included in the Brain Vision Analyzer software. Briefly, the imaging artifacts were automatically marked based on recurring patterns, the thus-defined intervals were averaged and their means subtracted from each interval. ECG pulse artifacts were removed by constructing an average ECG artifact template and subtracting it from the EEG data. Data were high-pass (0.5 Hz) and low-pass (70 Hz) filtered. Additionally, a notch filter was applied at 50 Hz. Using Independence Component Analysis (ICA), artifacts caused by eye movement, temporal electrode noise and residual pulse artifacts were removed. In case the electrode noise could not be eliminated by removing two independent components, the disturbed channel was removed and interpolated by topographical triangulation (occipital channels were not affected by this). After ICA, the data were again visually inspected for residual artifacts. No sleep patterns (i.e., K-complexes or sleep spindles) were present. EEG data from the AD group showed more artifacts such as eye movement and muscle activation, especially during the second half of the scan time, possibly constituting a sign of growing unrest. Two AD subjects showed a shift in frequency from alpha to theta over time. These artifacts were removed. The EEG signal was re-referenced to a common reference, obtained by averaging across all channels.

The electrodes O1, O2, and Oz were chosen as electrodes of interest, since alpha activity is best expressed at occipital electrodes (Moosmann et al., 2003; Laufs et al., 2003a; Mo et al., 2013). The arithmetic mean of electrophysiological activity from O1, O2, and Oz was calculated. Using complex demodulation, the EEG time courses of power within the total (8–12 Hz), lower (8–10 Hz), and upper (10–12 Hz) alpha band were extracted for each individual and exported to MATLAB (Mathworks, Inc., Sherborn, MA, United States) for the creation of statistical model regressors.

#### MRI Data

Functional magnetic resonance imaging data preprocessing was performed using SPM8^2^ implemented in Matlab 7 (Mathworks, Natick) and the VBM8 toolbox (Version 414^3^). The first six volumes were removed to eliminate saturation effects. Slices were referenced to the temporally middle slice. After realignment of the functional images, the anatomical images were coregistered to the realigned mean functional image. The structural T1-weighted MPRAGE images were segmented into gray matter, white matter and cerebrospinal fluid compartments and warped to standard MNI space, using the default MNI standard template and the Diffeomorphic Anatomical Registration Through Exponentiated Lie Algebra (DARTEL) method (Ashburner, 2007) implemented in VBM8. The resulting deformation fields were used to warp the functional images to standard space. Spatial smoothing of the normalized functional images was performed with a Gaussian Kernel of 8 mm full-width half-maximum (FWHM). In order to reduce slow drift artifacts, a high-pass filter with a cut-off period of 128 s was applied to the voxel time courses. From the segmented gray matter images, gray matter volume of the left and right hippocampus was calculated for each subject, using binarized inclusive masks that had been created for the IXI template in MNI space according to the international harmonization protocol for hippocampus segmentation (Grothe et al., 2012; Boccardi et al., 2015). The volume of the left and right hippocampus was pooled and normalized by dividing it by the total intracranial volume.

A regressor containing one-second intervals of artifact-free, averaged spectral power of the pooled occipital electrodes and an additional on/off regressor of no interest (containing timing information of artifacts longer than 1 s) were created. Separate regressors were built for power within the total alpha (8–12 Hz), lower alpha (8–10 Hz) and upper alpha band (10–12 Hz). The regressors of interest were convolved with an *a priori* defined hemodynamic response function (HRF) (Cohen, 1997) within the SPM first-level (single subject) processing pipeline (for a diagram see Supplementary Figure 1).

### Statistical Analysis

For comparing relative alpha band power at the pooled occipital channels (O1, O2, and Oz) between groups, Fast Fourier transformation (FFT) across 1-s-segments was used. Two-sided independent samples *t*-tests were used to compare relative alpha power and normalized hippocampal gray matter volume between groups. Separate general linear models were specified for total alpha, lower alpha and upper alpha, respectively, using SPM8 (Friston et al., 2007). The models included a regressor variable containing the power information for the respective HRF-convolved alpha band, a mean term regressor, a covariate regressor containing the artifact information, and the covariates age, gender, and years of education. For the first-level analysis, positive and negative t-contrasts were specified for each subject, testing for the effects of the alpha band power regressor, controlled for the artifact regressor. This resulted in individual statistical parametric maps of positive and of negative associations of the total, lower or upper alpha power fluctuation over time, respectively, with the BOLD fluctuation in each voxel of the brain. The resulting maps of EEG regressor weights were used for group comparisons in one- and two-sample *t*-tests. The one-sample *t*-tests were performed for the AD and HC group separately, testing for positive and negative associations of each alpha regressor weight across all subjects in the respective group. For the two-sample *t*-test, a contrast of HC > AD was defined for positive and negative associations, respectively. The second-level analyses were additionally controlled for the covariate regressor normalized hippocampal gray matter volume.

All statistical results were restricted to voxels within gray matter, by thresholding the default IXI template in VBM8 at *p* < 0.3 and using it as inclusive mask. Statistical significance levels were set at *p* < 0.01 (uncorrected for multiple comparisons). Only clusters with a voxel count ≥ 50 were considered. Resulting clusters were visually compared to a functional connectivity based DMN atlas (Shirer et al., 2012).

## Results

### Alpha Power Fluctuations

The mean relative alpha band power was not significantly different between the groups (AD: 35.0 ± 17.7%; HC: 32.0 ± 21.6%, Supplementary Table 1). However, at visual inspection, a morphological difference in the form of dysmorphic alpha waves was observed in the AD group.

### Association of Alpha Band Power and fMRI BOLD Dynamics

#### Positive Associations

**FIGURE 1:**
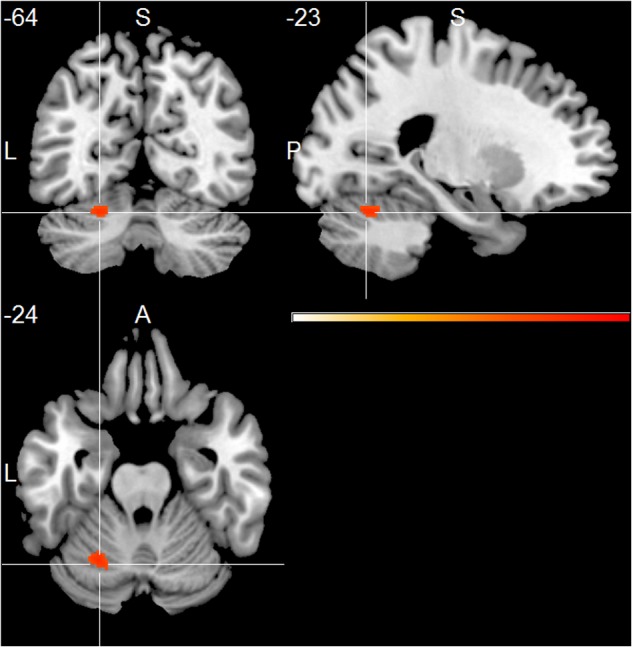
Alzheimer’s disease (AD) group effect, showing positive associations of total alpha band power fluctuation and BOLD signal (*p* < 0.01, uncorr., cluster threshold ≥ 50).

At group level, the AD group showed positive associations of total alpha band power with BOLD fluctuation in the cerebellum (one sample *t*-test, *p* < 0.01, uncorr., **Figure 1** and Supplementary Table 2). Lower alpha band power correlated positively with clusters in the right inferior temporal lobe, right hippocampus, left putamen and cerebellum (*p* < 0.01, uncorr.) (Supplementary Table 2). In contrast, power within the upper alpha frequency showed no significant positive associations in any regions.

**FIGURE 2:**
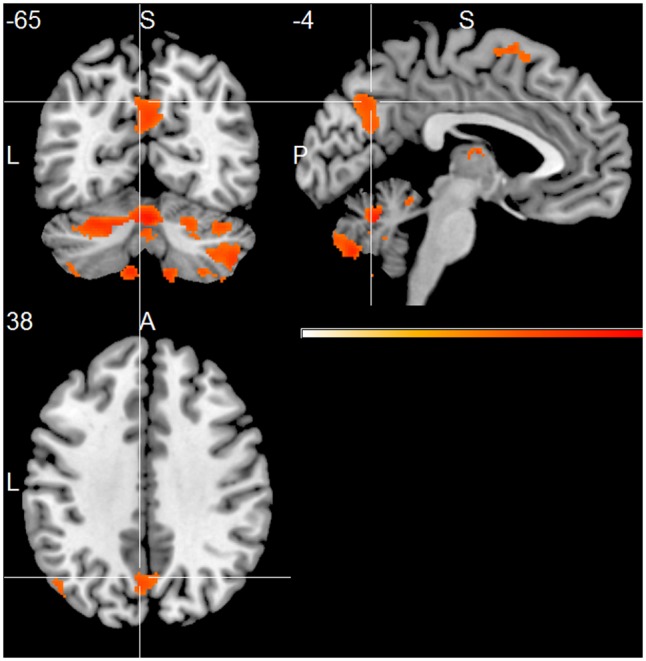
Healthy controls (HC) group effect, showing positive associations of total alpha band power fluctuation and BOLD signal (*p* < 0.01, uncorr., cluster threshold ≥ 50).

The HC group showed positive associations of total alpha band power with mainly frontal and temporal cortical regions, including superior, middle and inferior frontal cortex, temporal pole, parietal cortex, thalamus, putamen and cerebellum (one sample *t*-test, *p* < 0.01, uncorr., **Figure 2** and Supplementary Table 3). Within the lower alpha frequency, fewer associations were present, which were located mainly in frontal regions, left inferior temporal lobe, thalamus and cerebellum. Most associations were found within the upper frequency, located mainly in the hippocampus, thalamus, occipital, temporal and frontal cortex, including anterior cingulate cortex and middle cingulate, putamen and caudate nucleus, as well as cerebellum (Supplementary Table 3).

**FIGURE 3:**
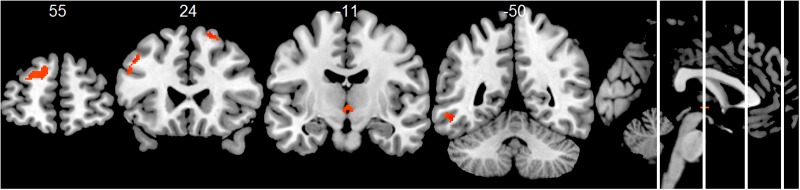
Group comparison HC > AD of positive associations of total alpha band power fluctuation and BOLD signal (*p* < 0.01, uncorr., cluster threshold ≥ 50).

**FIGURE 4:**
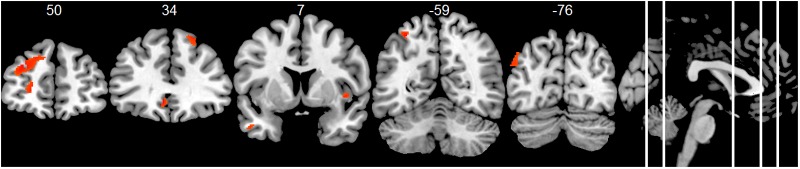
Group comparison HC > AD of positive associations of upper alpha band power fluctuation and BOLD signal (*p* < 0.01, uncorr., cluster threshold ≥ 50).

Compared to the HC group, the AD group showed significantly decreased positive associations of total alpha band power with BOLD fluctuation in clusters in the frontal cortex (superior, middle, inferior, precentral gyrus, and anterior cingulate cortex), inferior temporal lobe and thalamus (two-samples *t*-test, *p* < 0.01, uncorr., **Figure 3** and Supplementary Table 4). Similar decreased associations were found for the upper alpha band power (superior frontal lobe, insula and parietal lobe) (**Figure 4** and Supplementary Table 4). Regarding the lower alpha band power, the AD group showed decreased positive associations with scattered clusters in the superior frontal lobe, compared to the HC group (Supplementary Table 4).

At the individual level, first-level analyses revealed positive associations of power within the total alpha band range with regions that belong to the DMN (Shirer et al., 2012) in *n* = 6 HC subjects and in *n* = 3 AD patients (**Table 2**). For an example, see Supplementary Figures 2, 3.

**Table 2:** First-level analyses: number of subjects (*n*) showing positive associations of alpha band power and BOLD signal fluctuations, significant at *p* < 0.01 (uncorr.).

	Default mode network^∗^	Thalamus
**AD**		
Total alpha (8–12 Hz)	3	3
Lower alpha (8–10 Hz)	3	2
Upper alpha (10–12 Hz)	4	3
**HC**		
Total alpha (8–12 Hz)	6	5
Lower alpha (8–10 Hz)	4	3
Upper alpha (10–12 Hz)	7	5


*^∗^Encompassing three or more of the following regions: precuneus, PCC, ACC, medial prefrontal cortex, and inferior parietal lobe.*

Normalized hippocampal gray matter volume was lower in the AD group, although not significantly (independent samples *t*-test; *T*(26) = 1.735, *p* = 0.095). Entering it as covariate regressor in the general linear models did not essentially change the results of the one- and two-sample *t*-tests (Supplementary Figures 4–6).

#### Negative Associations

At group level, the AD group showed negative associations of total band alpha power with clusters in the occipital, frontal and temporal cortex (one-sample *t*-test, *p* < 0.01, uncorr., Supplementary Table 5). In the upper alpha band, associations were only significant in the occipital cortex. Lower alpha band power showed no significant associations (Supplementary Table 5).

The HC group showed significant negative associations of total alpha band power with clusters in the precentral gyrus and superior temporal cortex (one-sample *t*-test, *p* < 0.01, uncorr., Supplementary Table 6). No suprathreshold clusters were found in the upper alpha band. Lower alpha band power showed pronounced negative associations with the frontal cortex, mainly in the precentral and paracentral gyrus, and with the parietal cortex, temporal and middle cingulate cortex (Supplementary Table 6).

Compared to the HC group, the AD group did not exhibit significantly reduced negative associations of total or upper alpha band power with BOLD signal fluctuation in any voxel clusters. Regarding the lower alpha band, significantly decreased negative associations were found in the hippocampus, putamen and cerebellum (two-sample *t*-test, *p* < 0.01, uncorr., Supplementary Table 7).

At the individual level, first-level analyses revealed negative associations of alpha band power with BOLD fluctuations in both anterior and posterior regions in *n* = 5 AD patients and *n* = 7 HC subjects, associations in mainly frontal regions in *n* = 3 AD patients and *n* = 2 HC subjects, and associations in mainly posterior regions in *n* = 1 HC subject.

## Discussion

The study successfully applied simultaneous fMRI-EEG to an AD sample for the first time and showed a reduced positive association between alpha band power and BOLD fluctuations in the AD patients, compared to the HC subjects. In the HC group, positive associations between alpha band power and BOLD fluctuations were observed in numerous regions, including DMN regions. Although present in all alpha sub-bands, they were especially evident in the upper alpha frequency band. The reduction of these positive associations in the AD patients might be due to altered functional interaction between the brain regions (Greicius et al., 2004; Zhang et al., 2009, 2010; Agosta et al., 2012; Weiler et al., 2014; Xia et al., 2014). The functional associations were not altered by the correction for hippocampal volume, indicating that they were not driven by atrophy.

Based on previous simultaneous fMRI-EEG studies with healthy participants, we hypothesized to find a positive association of alpha band power and BOLD signal fluctuation in the thalamus in HC subjects (Goldman et al., 2002; Moosmann et al., 2003; Gonçalves et al., 2006). In the light of the disrupted integrity of the thalamo-cortical system, we expected this association to be reduced in the AD patients (Bhattacharya et al., 2011; Zhou et al., 2013). In line with the hypothesis, these associations were present in the HC group and were decreased in the AD group. Additionally, in both groups, we found more positive associations of the upper alpha band power with the thalamus compared to the lower alpha band. This might indicate a frequency-specificity. Also, as thalamo-cortical activity underlies alpha generation and modulation (Bhattacharya et al., 2013), future functional connectivity studies might investigate whether decreased associations of alpha band power and thalamic BOLD fluctuations are related to the thalamo-cortical connectivity in AD (Zhou et al., 2013).

The third hypothesis included finding negative associations with BOLD signal fluctuation in the occipital cortex. Negative associations were found at group level in AD patients in the occipital cortex, as well as superior medial frontal cortex and temporal cortex. However, we did not find negative associations with the occipital cortex in HC subjects at group level. This is in contrast to a number of fMRI-EEG studies in young healthy subjects, showing negative associations of alpha band power with BOLD signal in the occipital cortex (Goldman et al., 2002; Moosmann et al., 2003; Gonçalves et al., 2006; Mantini et al., 2007; Scheeringa et al., 2012). In the light of the overall accepted theory that alpha band represents a hallmark of the resting state of the brain (e.g., Gonçalves et al., 2006), we would have expected it to correlate negatively with BOLD signaling in the respective region. Instead, we found negative associations at HC group level in frontal, temporal and parietal regions. Although unexpected, this result is in line with a few other studies that reported an absence of negative associations with BOLD signal in the occipital cortex (Laufs et al., 2003a,b; Jann et al., 2009).

Interestingly, positive as well as negative associations with the cerebellum were present in almost all subjects. The cerebellum has received little attention in previous fMRI-EEG research (Scheeringa et al., 2012). FMRI studies showed impaired functional connectivity of the cerebellum in AD (Zheng et al., 2017), and a sensitivity of the cortico-cerebellar coupling to amyloid-β load in HC (Steininger et al., 2014). It would be interesting for future research to investigate the association of alpha band power and the integrity of cortical-cerebellar functional processes during rest.

A general limitation of fMRI resting state measurement is its high variability over time (Cole et al., 2010; Chen et al., 2015). The instruction to keep the eyes closed and to stay awake leaves room for spontaneous cognitive processes with varying attentional states. Possibly, the activation of the DMN might have been more robust if a task-based study design had been used, for example involving tasks of self-referential thinking or autobiographical memory (Andreasen et al., 1995; Mitchell, 2006; Gobbini et al., 2007; Spreng and Grady, 2010; Knyazev et al., 2011; Fomina et al., 2015). However, to be able to draw inferences on a potential clinical use, a resting state paradigm was needed. Another limitation is the relatively liberal statistical threshold. As this was the first study to employ simultaneous rsfMRI-rsEEG in AD patients, we aimed to assess the feasibility and to explore the associations in the whole brain.

We noted a high regional variability of both positive and negative associations between alpha band power fluctuation and BOLD signal between individual subjects, which has also been reported in previous studies (Goldman et al., 2002; Gonçalves et al., 2006; Laufs et al., 2006). Variability has been suggested to be partly caused by fluctuations in vigilance (Goldman et al., 2002; Laufs et al., 2006). Although our data were visually controlled for sleep, fluctuations in vigilance may have been present, particularly as an increase in artifacts in AD patients toward the end of the scan time was noted. The effect of vigilance on the association patterns of rsEEG and rsfMRI should be addressed in future research. Our results of high inter-individual heterogeneity, taken together with findings of high inter- and intra-individual variability observed in other resting state fMRI-EEG studies (Goldman et al., 2002; Laufs et al., 2003a, 2006; Moosmann et al., 2003; Gonçalves et al., 2006; Jann et al., 2009; Olbrich et al., 2009), also highlight the importance of future research with larger samples to be able to identify subgroups. Furthermore, our results support the necessity to differentiate the alpha band into sub-bands, as more HC subjects showed positive association patterns within the upper sub-band. This agrees with some other studies that investigated separate sub-bands (Laufs et al., 2006; Jann et al., 2009, 2010), linking sub-bands to different cognitive functions (e.g., Klimesch, 1999) and even indicating the possibility of predicting conversion from MCI to AD by calculating the ratio of power in alpha sub-bands (Moretti, 2015).

## Conclusion

The present study showed diminished positive associations between alpha band power fluctuation and BOLD signal fluctuations in several brain regions in AD patients, compared to HC subjects. These regions included (but were not limited to) DMN and thalamic regions. This study demonstrates the feasibility of measuring simultaneous rsEEG and rsfMRI signal fluctuations in a clinical AD population. Further research is needed to corroborate and expand its results.

## Author Contributions

KB recruited participants, performed neuropsychological testing, acquired EEG and MRI data, performed preprocessing and analyses, interpreted the data, drafted and revised the manuscript. CF recruited participants, conducted physical examinations, acquired EEG and MRI data, performed preprocessing and analyses, interpreted the data, and drafted the manuscript. CBe performed preprocessing and statistical analyses, interpreted the data, and revised the manuscript. SO contributed to the data interpretation and was involved in drafting the manuscript. CBa contributed to the study design, provided intellectual content for data interpretation, and revised the manuscript. ST was involved in all stages of the study, establishing the study design, recruiting participants, performing physical examinations, and revising the manuscript.

## Conflict of Interest Statement

The authors declare that the research was conducted in the absence of any commercial or financial relationships that could be construed as a potential conflict of interest.

## Footnotes

1 www.brainproducts.com

2 http://www.fil.ion.ucl.ac.uk/spm/

3 http://dbm.neuro.uni-jena.de/vbm8/
