# Supplementary material for: Early Changes in Alpha Band Power and DMN BOLD Activity in Alzheimer’s Disease: A Simultaneous Resting State EEG-fMRI Study
Source: Front Aging Neurosci. 2017 Oct 6;9:319. doi: 10.3389/fnagi.2017.00319 (PMC5635054; doi:10.3389/fnagi.2017.00319)
Supplement: Supplementary file 1 [file Data_Sheet_1.PDF]

Supplementary Material

Early changes in alpha band power and DMN BOLD activity in Alzheimer's disease: a simultaneous resting state EEG-fMRI study

Brueggen, Katharina[1]*, Fiala, Carmen[2]*, Berger, Christoph[3], Ochmann, Sina[2], Babiloni, Claudio.[4], & Teipel, Stefan[1,2]

*the authors contributed equally to the manuscript

Affiliations:

1) DZNE, German Center for Neurodegenerative Diseases, Rostock, Germany
2) Department of Psychosomatic Medicine, University Medicine Rostock, Rostock, Germany
3) Department of Psychiatry, Neurology, Psychosomatics, and Psychotherapy in Childhood and Adolescence, University Medical Center of Rostock, Rostock, Germany
4) Department of Physiology and Pharmacology "Vittorio Erspamer", University of Rome "La Sapienza" Rome, Italy; Department of Neuroscience, IRCCS San Raffaele Pisana, Rome, Italy

Corresponding author:

Katharina Brüggen
DZNE, German Center for Neurodegenerative Diseases, Rostock, Germany
Gehlsheimer Str. 20
18147 Rostock, Germany
Phone: +49 381 494 9478
Fax: +49 381 494 9472
Email: katharina.brueggen@dzne.de

1. Supplementary Figures and Tables

1.1. Supplementary Figures

Supplementary figure 1. Diagram showing the design of the general linear model using signal fluctuations of EEG and fMRI data

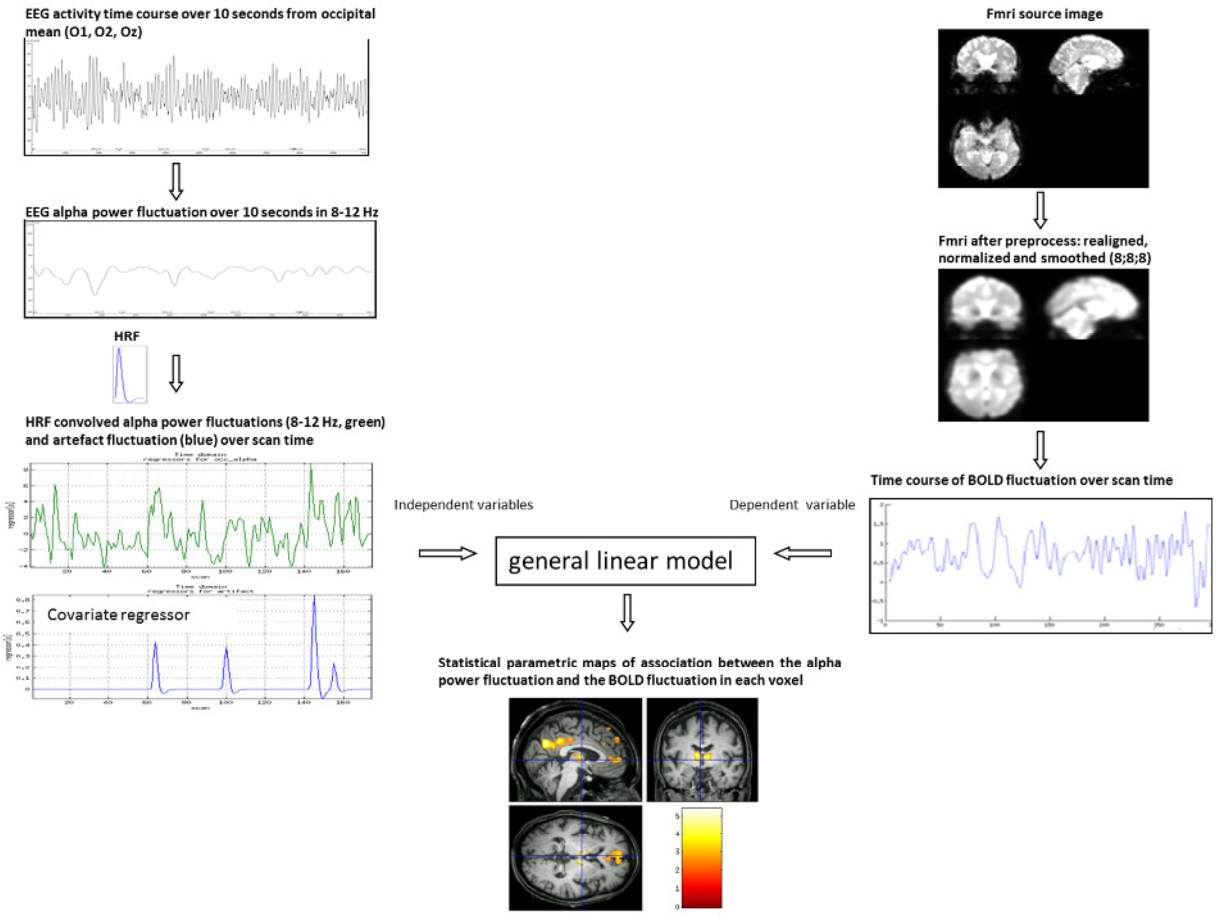

Supplementary figure 2. An AD subject showing positive associations of power within the total alpha band and BOLD signal ($p < 0.01$, uncorr., cluster size ≥ 50)

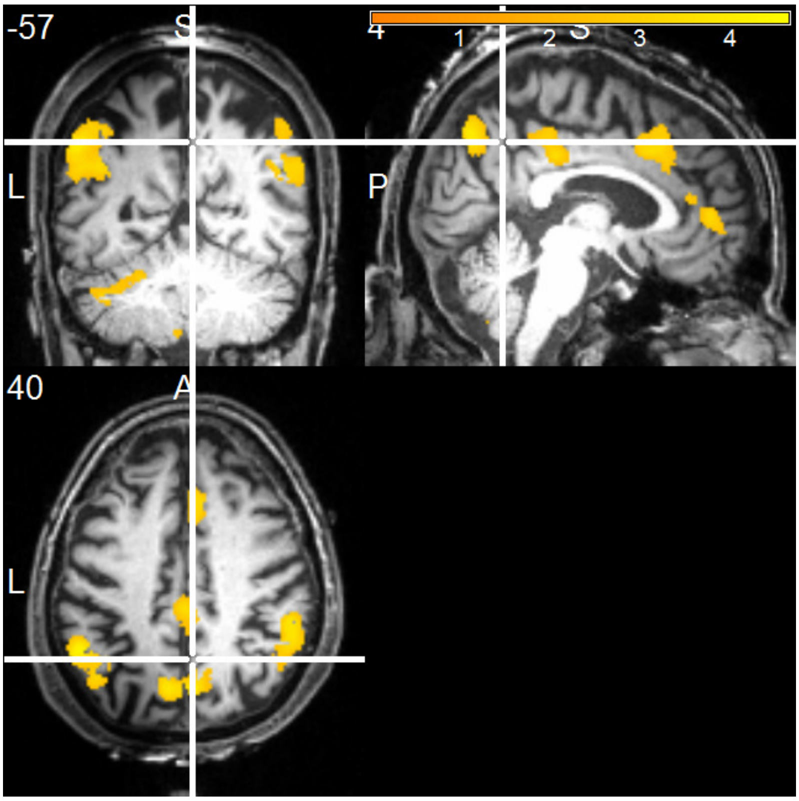

Supplementary figure 3. A HC subject showing positive associations of power within the total alpha band and BOLD signal ($p < 0.01$, uncorr., cluster size ≥ 50)

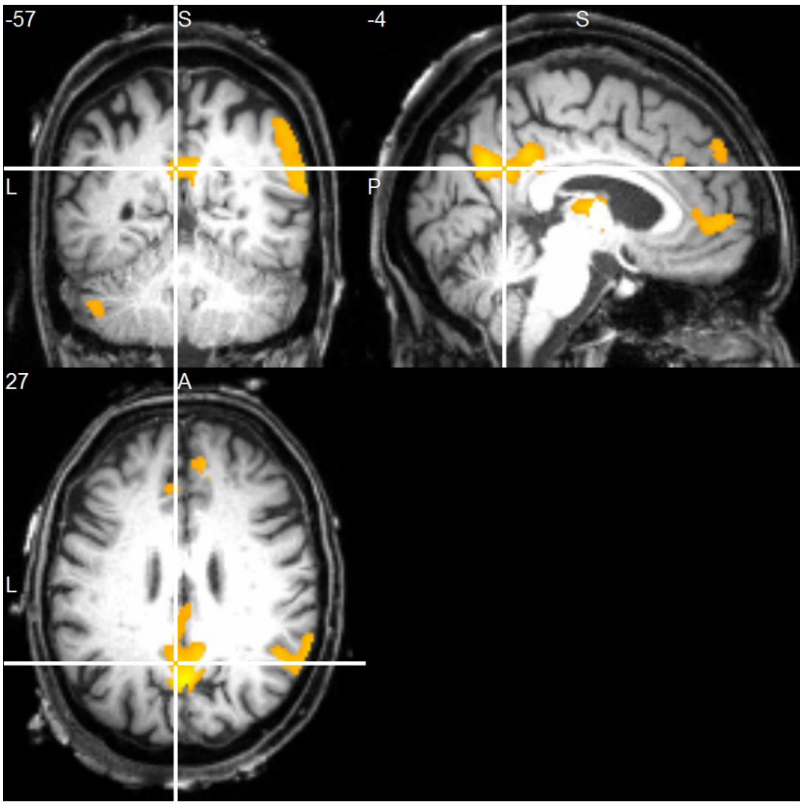

Supplementary figure 4. AD group effect, showing positive associations of total alpha band power fluctuation and BOLD signal, corrected for normalized hippocampal grey matter volume ($p < 0.01$, uncorr., cluster threshold ≥ 50)

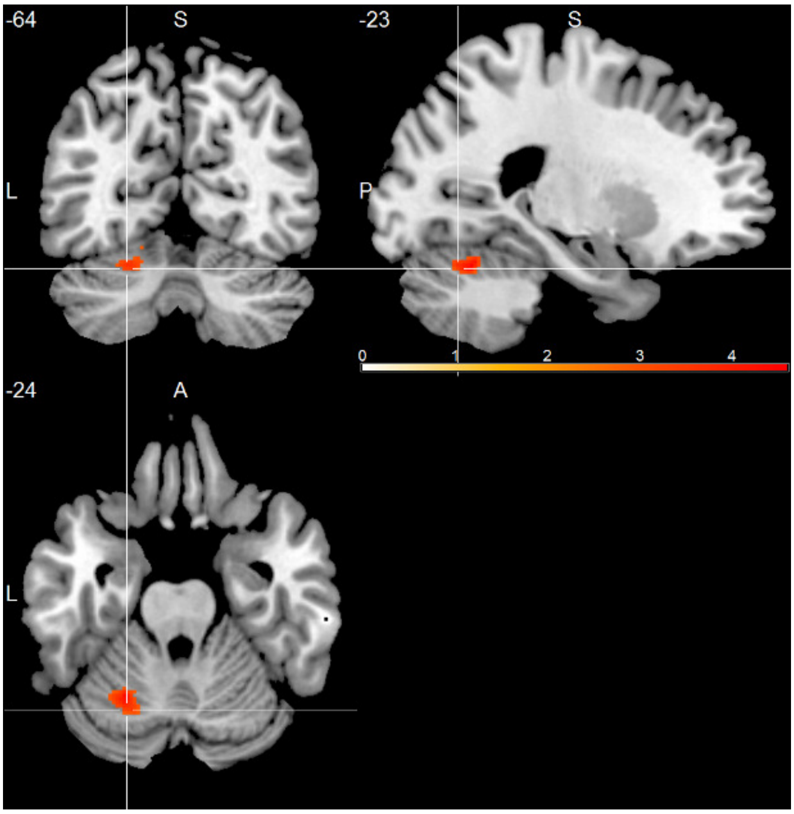

Supplementary figure 5. HC group effect, showing positive associations of total alpha band power fluctuation and BOLD signal, corrected for normalized hippocampal grey matter volume ($p < 0.01$, uncorr., cluster threshold ≥ 50)

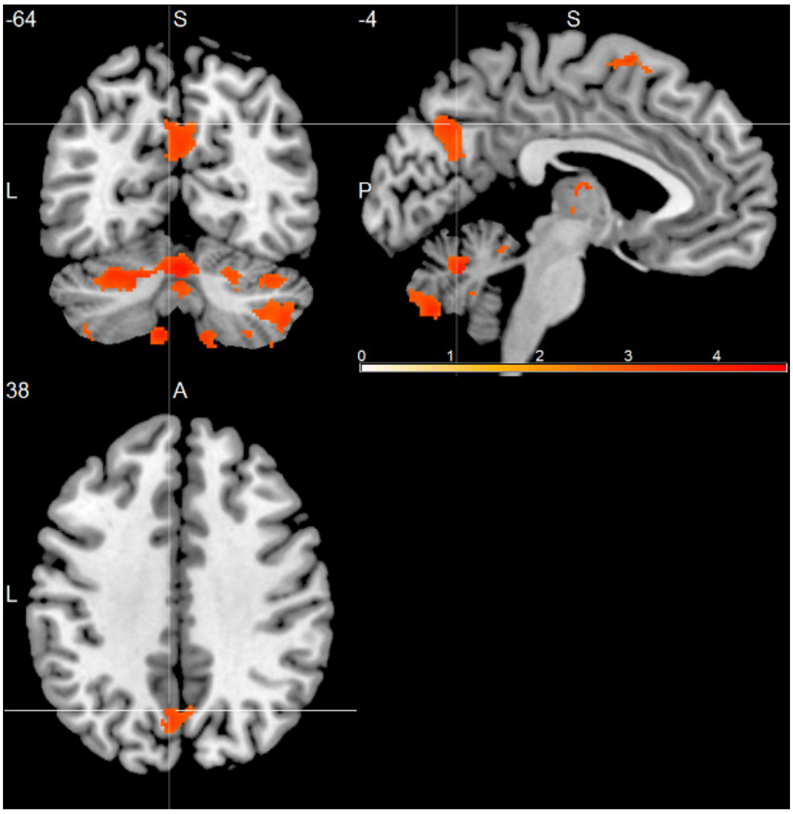

Supplementary figure 6. Group comparison HC > AD of positive associations of total alpha band power fluctuation and BOLD signal, corrected for normalized hippocampal grey matter volume ($p < 0.01$, uncorr. cluster threshold ≥ 50)

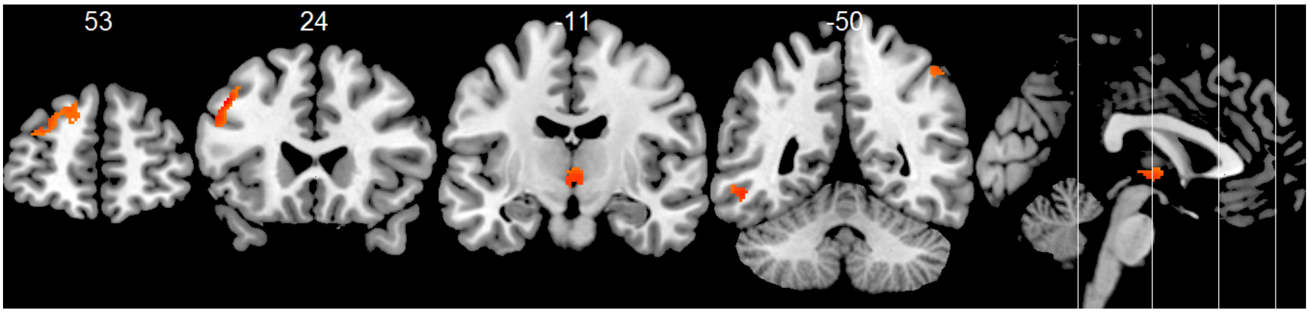

1.2. Supplementary tables

Supplementary Table 1. Relative power in total alpha band (8-12Hz); mean ± standard deviation (range)

Group	Relative power (%)	Artefacts (%)
AD	35.0 ± 17.7 (11.2 – 57.4)	11.1 ± 6.0 (0.9 – 22.3)
HC	32.0 ± 21.6 (8.8 – 92.6)	7.2 ± 4.7 (1.6 – 19.2)
p*	0.7	0.07

*independent samples t-test, 2-sided

Supplementary Table 2. Second-level analyses. Positive associations of alpha band power with BOLD fluctuations for the *AD group* (one-sample t-test, p < 0.01, uncorr., cluster size ≥ 50 voxels)

**Total alpha band (8-12 Hz)**						
**Region**	**Hemisphere**	**MNI coordinates (x y z)**			**Cluster size (voxels)**	**T (peak-level)**
Cerebellum	right	40	-45	-29	92	4.15
	left	-24	-61	-24	118	3.37
	left	-18	-67	-21	118	3.01
**Lower alpha band (8-10 Hz)**						
**Region**	**Hemisphere**	**MNI coordinates (x y z)**			**Cluster size (voxels)**	**T (peak-level)**
Temporal cortex Inferior	right	48	-49	-24	134	4.7
Hippocampus	right	16	-7	-17	144	4.19
	right	21	-13	-12	144	3.13
Putamen	left	-26	3	-8	51	3.16
Cerebellum	right	40	-44	-27	134	3.56
	left	-27	-60	-27	307	3.17
	left	-36	-58	-30	307	3.15
	left	-18	-67	-23	307	2.93
**Upper alpha band (10-12Hz)**						
No suprathreshold clusters						

Supplementary Table 3. Second-level analyses. Positive associations of alpha band power with BOLD fluctuations for the *HC group* (one-sample t-test, p < 0.01, uncorr., cluster size ≥ 50 voxels)

**Total alpha band (8-12 Hz)**							
**Region**		**Hemisphere**	**MNI coordinates (x y z)**			**Cluster size (voxels)**	**T (peak-level)**
Frontal cortex	Superior	right	24	60	0	259	3.85
		right	6	24	61	238	3.23
		right	18	24	61	238	3.18
		left	-21	48	-14	52	3.8
	Middle	right	40	45	-15	61	3.99
		right	38	60	6	259	3.49
		right	32	52	-5	52	3.32
		left	-33	47	-14	52	2.89
		left	-33	57	3	102	3.72
		left	-36	47	25	51	3.47
		left	-6	58	-6	33	3.31
		left	-42	17	43	77	3.19
	Inferior	left	-52	23	-2	88	3.38
		left	-42	21	-2	88	3.12
Supplementary motor cortex		right	4	6	61	238	3.66
		left	-4	9	64	115	3.34
Temporal cortex	Pole	left	-33	2	-44	271	3.56
	Superior	left	-40	-16	-9	136	3.34
		left	-40	-4	-12	136	3.12
	Middle	right	69	-31	-6	65	3.2
		right	68	-42	-8	65	3.13
		left	-60	-22	-14	222	4.09
		left	-54	-18	-23	222	3.82
		left	-66	-16	-12	222	3.24
		left	-60	-39	-9	133	2.8
	Inferior	left	-45	9	-36	271	4.5
		left	-52	-46	-12	133	3.96
		left	-33	-9	-35	50	3.23
	Insula	right	40	-19	0	149	3.33
Parietal cortex	Angular gyrus	left	-46	-70	40	54	3.44
Precuneus		left/right	0	-67	31	594	3.85
Thalamus		left	-4	-9	10	221	3.67
Putamen		right	36	-15	-6	149	3.62
Cerebellum		right	21	-85	-33	7602	5.03
		left	-2	-73	-41	7602	4.98
		left	-9	-66	-51	240	4.21
		left	-18	-73	-54	240	3.23
		left	-36	-60	-56	152	3.56
		left	-8	-57	-33	85	3.17
	Vermis	left/right	0	-63	-24	7602	4.89
	Vermis	right	3	-54	-36	85	3.47

Lower alpha band (8-10 Hz)							
**Region**		**Hemisphere**	**MNI coordinates (x y z)**			**Cluster size (voxels)**	**T (peak-level)**
Frontal cortex	Superior	right	22	60	3	76	4.19
		right	24	50	36	58	3.32
	Middle	right	42	57	-3	75	3.5
		right	34	48	30	58	3.42
Temporal cortex	Inferior	left	-44	9	-36	94	3.63
Thalamus		left	-2	-12	6	87	3.16
Cerebellum		right	24	-85	-36	1130	4.93
		right	27	-79	-44	1130	3.94
		right	33	-72	-44	1130	3.76
		right	6	-73	-47	100	3.96
		right	9	-63	-53	100	3.26
		left	-4	-75	-42	187	3.93
		left	-3	-82	-36	187	3.37
		left	-10	-43	-26	52	3.81
		left	-8	-61	-50	115	3.8
		left	-27	-67	-29	786	3.68
		left	-26	-81	-42	786	3.23
		left	-16	-67	-32	786	3.21
		left	-40	-58	-50	66	3.37
		Vermis	0	-64	-24	179	3.83

Upper alpha band (10-12 Hz)							
**Region**		**Hemisphere**	**MNI coordinates (x y z)**			**Cluster size (voxels)**	**T (peak-level)**
Frontal cortex	Superior	left	-18	60	9	1369	4.3
	Middle	right	46	12	49	56	3.57
		left	-30	59	4	1369	5.71
		left	-34	48	27	1369	5.52
		left	-8	50	-9	210	3.8
		left	-8	58	-6	210	3.52
	Inferior	right	54	21	-5	493	3.44
Precentral gyrus		left	-39	-7	54	57	3.98
Supplementary motor cortex		right	3	3	66	258	4.93
		left	-2	3	64	158	4.46
Cingulate cortex	Anterior	right	8	18	21	605	4.16
		left	-8	46	16	605	5.23
		left	-2	40	10	605	4.29
	Middle	right	9	-36	46	725	4.95
		left	-4	-33	45	725	4.73
		left	-4	0	36	102	4.12
Temporal cortex	Pole	right	57	10	-2	493	5.47
		left	-45	17	-18	1622	5.74
	Superior	right	66	-36	10	51	4.27
		right	46	-34	6	109	3.6
		left	-44	-27	6	2124	5.65
		left	-56	-30	16	2124	5.24
	Middle	right	62	-6	-15	289	7.52

Upper alpha band (10-12 Hz) (continued)

**Region**		**Hemisphere**	**MNI coordinates (x y z)**			**Cluster size (voxels)**	**T (peak-level)**
		right	58	0	-21	289	4.71
		right	69	-31	-6	145	4.87
		right	62	-28	-9	145	3.54
		right	54	-37	-5	109	3.38
		right	50	-40	1	109	3.13
		left	-51	-34	-8	842	6.15
		left	-62	-10	-11	842	5.83
		left	-56	-18	-17	842	5.08
		left	-39	-54	16	284	3.98
		left	-42	-64	21	284	3.16
	Inferior	left	-48	8	-36	1622	5.4
		left	-32	27	-11	1622	5.09
		right	63	-45	28	92	3.2
		right	60	-42	45	52	3.29
	Insula	right	44	-19	7	1305	6.51
		right	34	28	-3	66	4.02
		right	46	15	-5	493	4.85
		left	-33	-22	1	2124	5.58
Parietal cortex	Superior	right	36	-60	54	386	3.86
		left	-28	-64	55	88	3.99
	Inferior	right	36	-54	43	386	4.69
	Supramarginal gyrus	right	66	-37	28	92	3.61
	Postcentral gyrus	right	63	-18	15	1305	5.4
	Angular gyrus	right	30	-64	46	386	3.61
Occipital cortex	Middle	right	56	-64	24	52	3.43
		right	42	-64	22	52	3.3
		left	-48	-73	30	284	3.17
	Lingual cortex	left	-8	-45	1	59	3.73
Parahippocampal gyrus		right	20	-10	-30	78	6.29
Hippocampus		left	-14	-13	-20	18984	9.74
		left	-33	-36	-9	60	5.06
Putamen		right	36	-15	0	1305	7.31
Caudate nucleus		right	12	2	9	73	4.27
		left	-18	5	16	53	3.22
		left	-15	8	9	53	2.92
		left	-16	-16	21	41	3.24
Thalamus		right	16	-30	1	199	4.86
		left	-2	-13	12	669	6.38
		left/right	0	-6	6	669	4.77
		left	-9	-3	10	53	4.67
Cerebellum		left	-9	-49	-17	18984	9.39
		left	-18	-55	-54	1248	6.75
		left	-32	-60	-54	1248	5.42

Upper alpha band (10-12 Hz) (continued)

Region		Hemisphere	MNI coordinates (x y z)			Cluster size (voxels)	T (peak-level)
		left	-14	-42	-47	1248	4.16
	Vermis	right	6	-60	-23	18984	9.3

Supplementary Table 4. Second-level analyses (HC > AD): Positive associations of alpha band power and BOLD fluctuations (two-sample t-test, $p < 0.01$, uncorr., cluster size ≥ 50 voxels)

Total alpha band (8-12Hz)

Region		Hemisphere	MNI coordinates (x y z)			Cluster size (voxels)	T (peak-level)
Frontal lobe	Superior	right	20	26	60	61	3.44
		left	-16	59	24	166	3.06
		left	-15	53	33	166	2.65
	Middle	left	-45	24	39	140	3.38
		left	-26	53	28	166	2.85
	Inferior	left	-50	29	28	140	3.63
Temporal lobe	Inferior	left	-54	-48	-11	71	3.36
Thalamus		left	3	-15	-5	65	2.93
**Lower alpha band (8-10Hz)**							
**Region**		**Hemisphere**	**MNI coordinates (x y z)**			**Cluster size (voxels)**	**T (peak-level)**
Frontal lobe	Superior	left	-14	54	25	81	3.05
**Upper alpha band (10-12Hz)**							
**Region**		**Hemisphere**	**MNI coordinates (x y z)**			**Cluster size (voxels)**	**T (peak-level)**
Frontal cortex	Superior	right	22	33	52	78	3.07
		right	26	24	54	78	2.7
		left	-16	53	33	528	3.24
		left	-24	56	25	528	3.18
		left	-22	50	3	205	3.28
		left	-18	62	3	205	3.83
		left	-20	39	40	139	3.18
		left	-24	14	46	56	2.73
		left	-32	51	25	528	3.76
		left	-30	59	4	205	3.33
		left	-26	23	46	56	2.79
Temporal cortex	Insula	right	44	6	-3	50	3.41
Parietal lobe	Superior	left	-33	-58	55	75	3.44
	Angular gyrus	left	-40	-78	39	80	2.92
		left	-45	-76	30	80	2.97

Supplementary Table 5. Second-level analyses: Negative associations of alpha band power with BOLD fluctuations for the *AD group* (one-sample t-test, $p < 0.01$, uncorr.)

**Total alpha band (8-12 Hz)**							
**Region**		**Hemisphere**	**MNI coordinates (x y z)**			**Cluster size (voxels)**	**T (peak-level)**
Frontal cortex	Superior	left	-12	57	27	98	3.54
		left	-21	56	28	98	2.88
	Middle	right	4	40	-12	137	3.17
		right	8	34	-18	137	2.93
Temporal cortex	Pole	right	48	14	-17	186	4.48
		right	51	9	-23	186	3.66
Occipital cortex	Superior	right	22	-85	34	364	4.35
		right	20	-87	24	364	3.31
		right	24	-82	42	364	3.26
	Middle	left	-27	-88	27	74	3.22
	Lingual gyrus	left	-32	-88	-14	116	5.45
		left	-14	-88	-17	116	3.48
	Cuneus	left	-8	-90	22	326	4.72
		left	-2	-82	27	326	4.51
		left	-6	-85	33	326	3.64
Cingulate cortex	Anterior	left	-6	33	-6	137	3.5
**Lower alpha band (8-10 Hz)**							
No suprathreshold clusters							
**Upper alpha band (10-12Hz)**							
**Region**		**Hemisphere**	**MNI coordinates (x y z)**			**Cluster size (voxels)**	**T (peak-level)**
Occipital lobe	Superior	left	-22	-81	33	90	3.35
	Middle	left	-24	-82	24	90	2.97

Supplementary Table 6. Second-level analyses: Negative associations of alpha band power with BOLD fluctuations for the *HC group* (one-sample t-test, $p < 0.01$, uncorr.)

**Total alpha band (8-12 Hz)**							
**Region**		**Hemisphere**	**MNI coordinates (x y z)**			**Cluster size (voxels)**	**T (peak-level)**
Precentral gyrus		right	28	-7	51	57	3.68
		right	26	-24	60	20	3.31
		left	-46	5	18	102	4.32
Temporal cortex	Superior	right	58	3	-14	71	3.88
**Lower alpha band (8-10 Hz)**							
**Region**		**Hemisphere**	**MNI coordinates (x y z)**			**Cluster size (voxels)**	**T (peak-level)**
Frontal cortex	Superior	left	-21	-1	55	222	4.47
		left	-2	17	42	112	3.95
	Inferior	right	34	8	33	118	4.26
		right	40	9	25	118	2.85
		left	-45	8	13	87	3.53
		left	-38	9	24	87	3.19
Supplementary motor cortex		right	9	6	49	200	3.73
		left	3	15	43	123	3.8

Precentral gyrus		left	-39	-1	39	56	3.25
Paracentral gyrus		right	8	-31	52	71	3.46
		left	-3	-27	49	146	4.1
		left	-6	-24	58	146	3.14
Temporal cortex	Superior	right	58	2	-12	197	4.1
	Middle	right	64	-1	-18	197	3.02
	Insula	right	36	-12	15	112	4.16
		right	44	-4	15	112	3.61
Parietal cortex	Inferior	left	-33	-40	43	94	3.45
		left	-36	-31	42	94	3.23
Cingulate cortex	Middle	right	8	-4	40	200	3.99
		right	12	18	37	123	3.16
**Upper alpha band (10-12Hz)**							
No suprathreshold clusters							

Supplementary Table 7. Second-level analyses (HC > AD): Negative associations of alpha band and BOLD fluctuations (two-sample t-test, p < 0.01, uncorr.)

**Total alpha band (8-12Hz)**							
No suprathreshold clusters							
**Lower alpha band (8-10 Hz)**							
**Region**		**Hemisphere**	**MNI coordinates (x y z)**			**Cluster size (voxels)**	**T (peak-level)**
Hippocampus		right	16	-9	-17	64	3.1
Putamen		left	-27	5	-8	194	4.25
		left	-24	12	0	194	2.93
Cerebellum		left	-24	-58	-26	55	3.19
		left	-6	-52	-15	140	2.85
	Vermis	right	4	-46	-14	140	3.17
**Upper alpha band (10-12 Hz)**							
No suprathreshold clusters							
